# Assessing Physicians’ and Nurses’ Awareness of Health Educators’ Roles and Its Impact on Patient Experience in Healthcare Settings: A Cross-sectional Study in Saudi Arabia

**DOI:** 10.7759/cureus.86352

**Published:** 2025-06-19

**Authors:** Nawaf Alnuwaysir, Bayader Alotaiby, Lujain Bin Amer, Atheer Al Dera, Reem Alsaeed

**Affiliations:** 1 Community Health Sciences, King Saud University, Riyadh, SAU; 2 Directorate of Studies and Change Management, Ministry of Health, Riyadh, SAU; 3 College of Public Health, King Saud Bin Abdulaziz University for Health Sciences, Riyadh, SAU; 4 Health Education, King Khalid University Hospital, Riyadh, SAU; 5 College of Health and Rehabilitation Sciences, Princess Nourah Bint Abdulrahman University, Riyadh, SAU

**Keywords:** awareness, health education, nurses, patient experience, physicians, saudi arabia

## Abstract

Background: Health educators play a crucial role in improving patient outcomes by fostering communication, enhancing treatment adherence, and promoting health education. However, limited awareness of their roles among healthcare professionals may hinder interdisciplinary collaboration and the integration of health education in clinical settings.

Objective: This study aimed to assess the awareness of physicians and nurses regarding the roles of health educators and examine their impact on patient experience in Saudi Arabian healthcare settings.

Methods: A cross-sectional survey was conducted among 192 physicians and nurses across various healthcare facilities. Data were collected using a structured questionnaire assessing knowledge, awareness, and perceived importance of health educators. Descriptive and inferential statistical analyses, including chi-square tests, were performed to examine associations between awareness levels and demographic characteristics.

Results: Findings indicate that 69.27% of participants demonstrated high awareness of health educators' roles, while 22.92% exhibited limited understanding of their presence in healthcare settings. Awareness levels were significantly associated with years of experience. Additionally, 90.10% of participants acknowledged the critical role of health educators in enhancing patient experience, emphasizing the need for their structured integration into healthcare teams.

Conclusion: Although healthcare professionals generally acknowledge the importance of health educators, gaps in awareness, presence, and roles persist. To address these gaps, healthcare institutions should integrate structured training programs and interprofessional education to enhance collaboration among healthcare professionals, thereby improving patient outcomes.

## Introduction

The World Health Organization (WHO) defines health education as a set of experiences aimed at improving individual and community well-being. Health educators play a vital role in this effort, with responsibilities including assessing needs, planning, implementing, and evaluating health programs, coordinating services, and communicating health-related information [1]. Health educators work in various settings, primarily hospitals, focusing on patient education, health promotion, prevention, and advocacy. Health educators work with physicians and nurses to improve the care and well-being of patients. 

Patient education helps reduce readmissions by ensuring patients understand discharge instructions and manage their conditions effectively [2]. In Saudi Arabia, health education gained official recognition in 2004 when the Saudi Commission for Health Specialties (SCFHS) classified it as a distinct health profession [3]. This highlights the critical role that health educators play in raising public health awareness and enhancing community health.

The level of awareness among healthcare professionals regarding health educators' roles is influenced by several factors. These include educational background, experience working with health educators, and the institutional culture of the hospital. Clarifying roles in primary care is a shared responsibility between the organization and the members of the primary care team [4]. 

Despite their role in improving patient outcomes, studies show that many physicians and nurses lack a full understanding of health educators' contributions to the patients. For instance, a study in Saudi Arabia found that 48.41% of healthcare professionals had only a fair level of awareness of health educators' roles, with many mistakenly believing they perform tasks like wound care and checking vital signs [5]. Also, a study shows that healthcare workers in Saudi Arabia view health education as an important part of their practice and calls for targeted efforts to strengthen health education in primary centers [6].

Patient experience includes interactions with healthcare providers and healthcare staff across settings. As a key aspect of care quality, it reflects what matters most to patients, such as timely appointments, clear access to information, and good communication [7].

The Consumer Assessment of Healthcare Providers and Systems (CAHPS) surveys are key tools used to evaluate patient-centered care. These surveys capture patients’ perspectives on care using standardized and validated questions, allowing providers and stakeholders to identify areas for improvement [7]. In Saudi Arabia, the Ministry of Health administers Press Ganey surveys quarterly to monitor patient experience outcomes, particularly complaints. This reflects a national effort to continuously improve healthcare services in alignment with patient needs and expectations [8].

Improving awareness of health educators' roles among healthcare workers through training programs and interdisciplinary collaboration or meetings can lead to better patient outcomes and understanding of the importance of their roles. Considering this, this study seeks to assess the level of knowledge and understanding among physicians and nurses regarding the roles of health educators. Despite the recognized importance of health educators in improving patient health outcomes, there is a noticeable lack of research exploring how well physicians and nurses understand the roles and responsibilities of health educators in Saudi Arabia. This study aims to fill that gap by (i) assessing the current level of knowledge and understanding among physicians and nurses regarding the roles of health educators and (ii) examining the importance of health educators’ responsibilities in improving patient well-being.

## Materials and methods

Study design

A cross-sectional design study survey was conducted in Saudi Arabia between February and July 2024. The study targeted male and female physicians and nurses working in public and private hospitals, as well as other healthcare settings across the country.

Sample technique and sample size**

The study utilized a convenient non-probability sampling method, relying on the accessibility and willingness of participants to complete the survey. Readily accessible physicians and nurses were selected to facilitate data collection and improve response rates. The final sample included 192 participants. As of 2023, the healthcare workforce in Saudi Arabia comprises approximately 113,300 physicians and 218,107 nurses working across various healthcare settings [9].

Inclusion and exclusion criteria

Participants included in the study were currently employed nurses and physicians in Saudi Arabia, working in healthcare settings, who were at least 18 years of age, and provided informed consent before participation. The study excluded 36 participants from the original 228 responses who either refused to agree to the informed consent, were students, or were under the age of 18, while all other eligible respondents were invited to take part. 

Data collection tool and instrument**

A questionnaire was developed using the online survey tool Google Forms (Google, Mountain View, CA, US), and it was distributed through popular social media platforms (e.g., WhatsApp, LinkedIn, and X) as well as the help from the Saudi Commission for Health Specialties (SCFHS) distribution service. The questionnaire consisted of 33 questions, divided into four sections: sociodemographic characteristics, knowledge of health education, knowledge of the presence of health educators, and knowledge of the tasks, roles, and responsibilities of health educators. The instrument utilized a three-point Likert scale, and all questions were mandatory to answer in order not to have missing data. The questionnaire took approximately 2-4 minutes to complete. 

Validity and reliability

To ensure the validity of our data collection tool, we conducted a pilot test of ~30 participants and sought feedback from experts in the field of health education. The pilot test allowed us to identify any potential issues with the tool’s clarity and functionality, while the expert review provided an additional layer of validation regarding the tool’s relevance and accuracy. Both methods confirmed that the tool was valid and suitable for capturing the intended data. Cronbach’s α was done to test the reliability, which confirms the internal consistency of the questions, with a score of 0.773 indicating acceptable reliability.

Ethical considerations 

The survey was designed to be completed anonymously, ensuring privacy and reducing social desirability bias. Ethical approval for this study was obtained from the Institutional Review Board (IRB) of King Saud University (Approval Number: 25-470). The study adhered to the ethical principles outlined in the Declaration of Helsinki, ensuring participant confidentiality and voluntary participation. Informed consent was obtained electronically before participants could proceed with the survey.

Statistical analysis

Microsoft Excel (Microsoft Corp., Redmond, WA, US) was utilized for data management, while JMP Pro 18 (JMP Statistical Discovery LLC, Cary, NC, US) was used for statistical analysis. Tables and figures were used to present descriptive data. To compare the relationship between knowledge and reported knowledge, as well as the association between knowledge and sociodemographic characteristics, a chi-square test was conducted. This statistical test helped in assessing the significance of the observed differences across the variables. Participants were prompted, through an open-ended question, to report their medical specialty. Then, each response was categorized into one of eight groups: six major specialty categories adapted from a previous study [10], which based its classification on the European Union of Medical Specialists (UEMS)' list of specialties, and the two additional categories "other" and "not mentioned" were introduced in our study. Moreover, the scores for the two types of knowledge (presence of health educators’ knowledge and tasks, roles, and responsibilities of health educators' knowledge) were calculated and subsequently classified into three levels: low, moderate, and high. Any p-value below 0.05 was considered statistically significant.

## Results

Background information of participants

A total of 192 physicians and nurses participated in the study, with the majority being physicians (n = 121, 63.02%). Participants predominantly fell within the 26-30 age range (n = 58, 30.21%), and a slightly higher proportion were female (n = 104, 54.17%). Most had less than five years of experience (n = 101, 52.60%) and held a bachelor’s degree (n = 98, 51.04%). Additionally, 144 participants (75.00%) resided in the central region of Saudi Arabia (Table 1).

**Table 1:** Frequency distribution of sociodemographic characteristics (single response)

Characteristics	N	% of total
Gender		
Female	104	54.17%
Male	88	45.83%
Age		
18-20 years old	5	2.60%
21-25 years old	36	18.75%
26-30 years old	58	30.21%
31-35 years old	33	17.19%
36-40 years old	29	15.10%
41-45 years old	13	6.77%
46-50 years old	4	2.08%
51-55 years old	7	3.65%
56 years and older	7	3.65%
Profession		
Nurse	71	36.98%
Physician	121	63.02%
Years of experience		
Over 15 years	27	14.06%
11-15 years	18	9.38%
5-10 years	46	23.96%
Less than 5 years	101	52.60%
Educational level		
Fellowship	36	18.75%
Residency	22	11.46%
Master’s degree	22	11.46%
Bachelor’s degree	98	51.04%
Diploma	14	7.29%
Region		
Eastern region	15	7.81%
Middle region	144	75.00%
Northern region	8	4.17%
Southern region	5	2.60%
Western region	20	10.42%

In terms of specialty, almost half specialized in internal medicine (n = 98, 47.80%), with their workplaces fairly split between outpatient clinics (n = 74, 35.75%) and inpatient departments (n = 74, 35.75%) (Table 2).

**Table 2:** Frequency distribution of sociodemographic characteristics (multiple responses) Note: Participants could select more than one option; therefore, totals reflect the number of responses, not the number of participants. “Other” in the "Specialty" section includes nursing technician, clinical instructor, dentistry, midwifery, clinical nutrition, and pharmacology. “Other” in the "Usual place of work" section includes the surgery department, delivery room, infection control department, radiology department, pharmacy, Ministry of Health, primary care center, and nursing college.

Characteristics	N	% of total
Specialty		
Surgery	19	9.27%
Diagnostic	4	1.95%
Public health	11	5.37%
Psychiatry	1	0.49%
Anesthesiology and emergency	7	3.41%
Internal medicine	98	47.80%
Other	21	10.24%
Did not mention	44	21.46%
Total	205	100.00%
Usual place of work		
Outpatient clinic	74	35.75%
Inpatient department	74	35.75%
Emergency room	25	12.08%
Intensive care unit	5	2.42%
Hospital administration	6	2.90%
Other	14	6.76%
Did not mention	9	4.35%
Total	207	100.00%

Comparison of participants’ awareness and reported knowledge

There was no significant association between participants' knowledge of the presence of health educators and their awareness levels (χ² = 3.240, p > 0.05, Table 3). However, a significant association was found between their understanding of health educators' roles and responsibilities and their reported knowledge (χ² = 8.002*, p < 0.05, Table 4).

**Table 3:** Chi-square analysis of knowledge of health educators’ presence vs. perceived knowledge Note: Percentages are based on column totals. χ²(2) = 3.240, p = 0.1979.

Do you know about health education?	High	Moderate	Low	Total
Yes	74 (92.50%)	59 (86.76%)	36 (81.82%)	169 (88.02%)
No	6 (7.50%)	9 (13.24%)	8 (18.18%)	23 (11.98%)
Total	80 (41.67%)	68 (35.42%)	44 (22.92%)	192 (100.00%)

**Table 4:** Chi-square analysis of knowledge of health educators’ roles and responsibilities vs. perceived knowledge Note: Percentages are based on column totals. χ²(2) = 8.002, p = 0.0183*.

Do you know about health education?	High	Moderate	Low	Total
Yes	117 (87.97%)	36 (97.30%)	16 (72.73%)	169 (88.02%)
No	16 (12.03%)	1 (2.70%)	6 (27.27%)	23 (11.98%)
Total	133 (69.27%)	37 (19.27%)	22 (11.46%)	192 (100.00%)

Profession and health educator interaction

No significant relationship was found between the participants’ profession (physician or nurse) and their experience working with health educators (χ² = 0.576, p > 0.05, Table 5).

**Table 5:** Association between profession and experience working with health educators Note: Percentages are based on row totals. χ²(2) = 0.576, p =0.7498.

Profession	Yes	No	I don’t remember	Total
Nurse	37 (52.11%)	25 (35.21%)	9 (12.68%)	71 (36.98%)
Physician	62 (51.24%)	39 (32.23%)	20 (16.53%)	121 (63.02%)
Total	99 (51.56%)	64 (33.33%)	29 (15.10%)	192 (100.00%)

Awareness and years of experience

Participants’ years of experience were not significantly associated with knowledge of health educators’ presence (χ² = 10.480, p > 0.05, Table 6). However, there was a significant association between years of experience and understanding of health educators' roles, tasks, and responsibilities (χ² = 22.797*, p < 0.01, Table 7).

**Table 6:** Association between profession and experience working with health educators Note: Percentages are based on row totals. χ²(6) = 10.480, p =0.1059.

Years of experience	High	Moderate	Low	Total
Over 15 years	17 (62.96%)	7 (25.93%)	3 (11.11%)	27 (14.06%)
11-15 years	10 (55.56%)	3 (16.67%)	5 (27.78%)	18 (9.38%)
5-10 years	17 (36.96%)	18 (39.13%)	11 (23.91%)	46 (23.96%)
Less than 5 years	36 (35.64%)	40 (39.60%)	25 (24.75%)	101 (52.60%)
Total	80 (41.67%)	68 (35.42%)	44 (22.92%)	192 (100.00%)

**Table 7:** Association between years of experience and knowledge of health educators’ tasks, roles, and responsibilities Note: Percentages are based on row totals. χ²(6) = 22.797, p =0.0009*.

Years of experience	High	Moderate	Low	Total
Over 15 years	15 (55.56%)	11 (40.74%)	1 (3.70%)	27 (14.06%)
11-15 years	8 (44.44%)	3 (16.67%)	7 (38.89%)	18 (9.38%)
5-10 years	39 (84.78%)	4 (8.70%)	3 (6.52%)	46 (23.96%)
Less than 5 years	71 (70.30%)	19 (18.81%)	11 (10.89%)	101 (52.60%)
Total	133 (69.27%)	37 (19.27%)	22 (11.46%)	192 (100.00%)

Participants’ awareness

**Figure 1:**
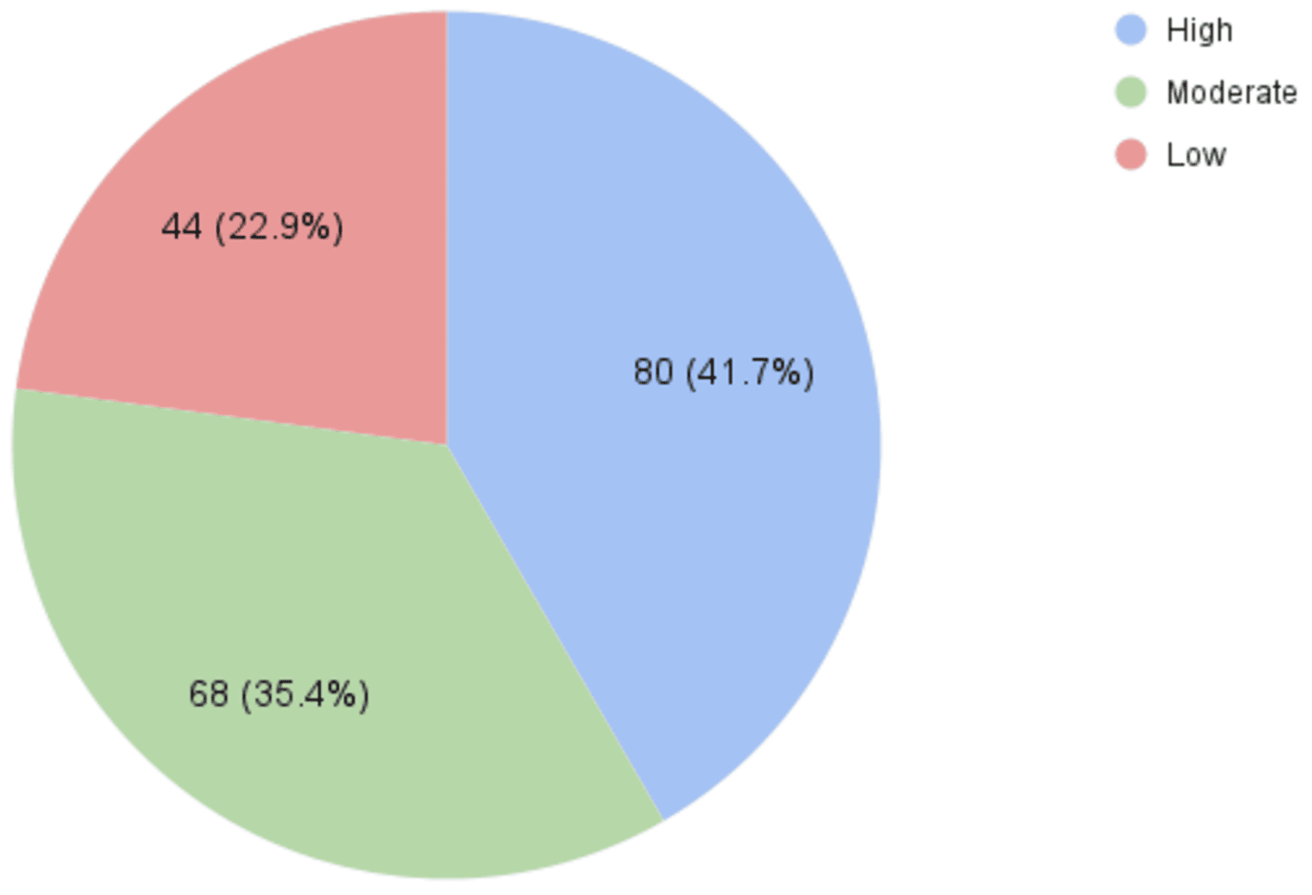
Knowledge levels on the presence of health educators (high, moderate, low)

**Figure 2:**
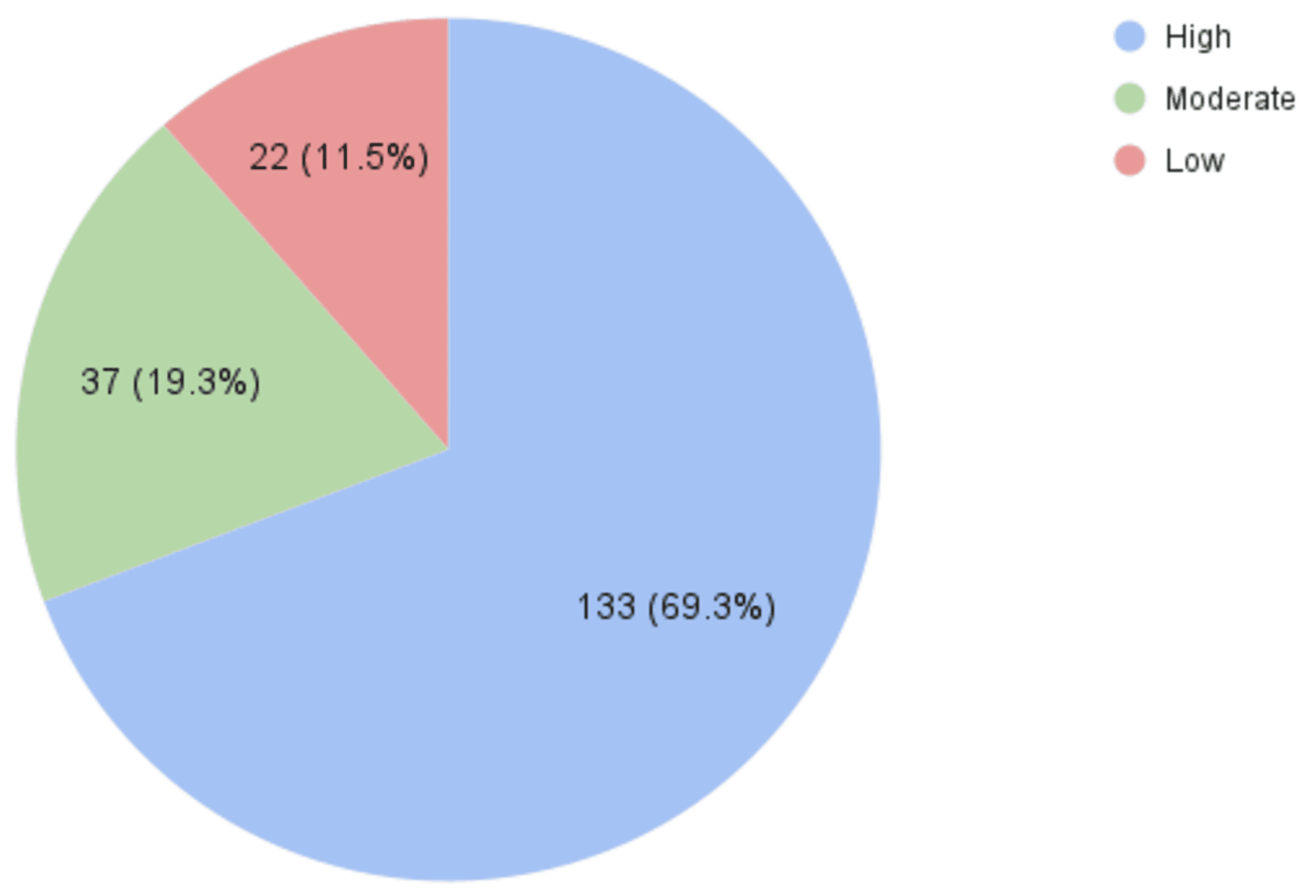
Knowledge levels on health educators’ tasks, roles, and responsibilities (high, moderate, low)

A larger group (n = 44, 22.9%) demonstrated low awareness of the presence of health educators (Figure 1), while only 22 (11.5%) participants scored low on their understanding of health educators’ tasks, roles, and responsibilities (Figure 2). Notably, 133 (69.3%) participants showed high levels of understanding regarding health educators' roles (Figure 2).

**Figure 3:**
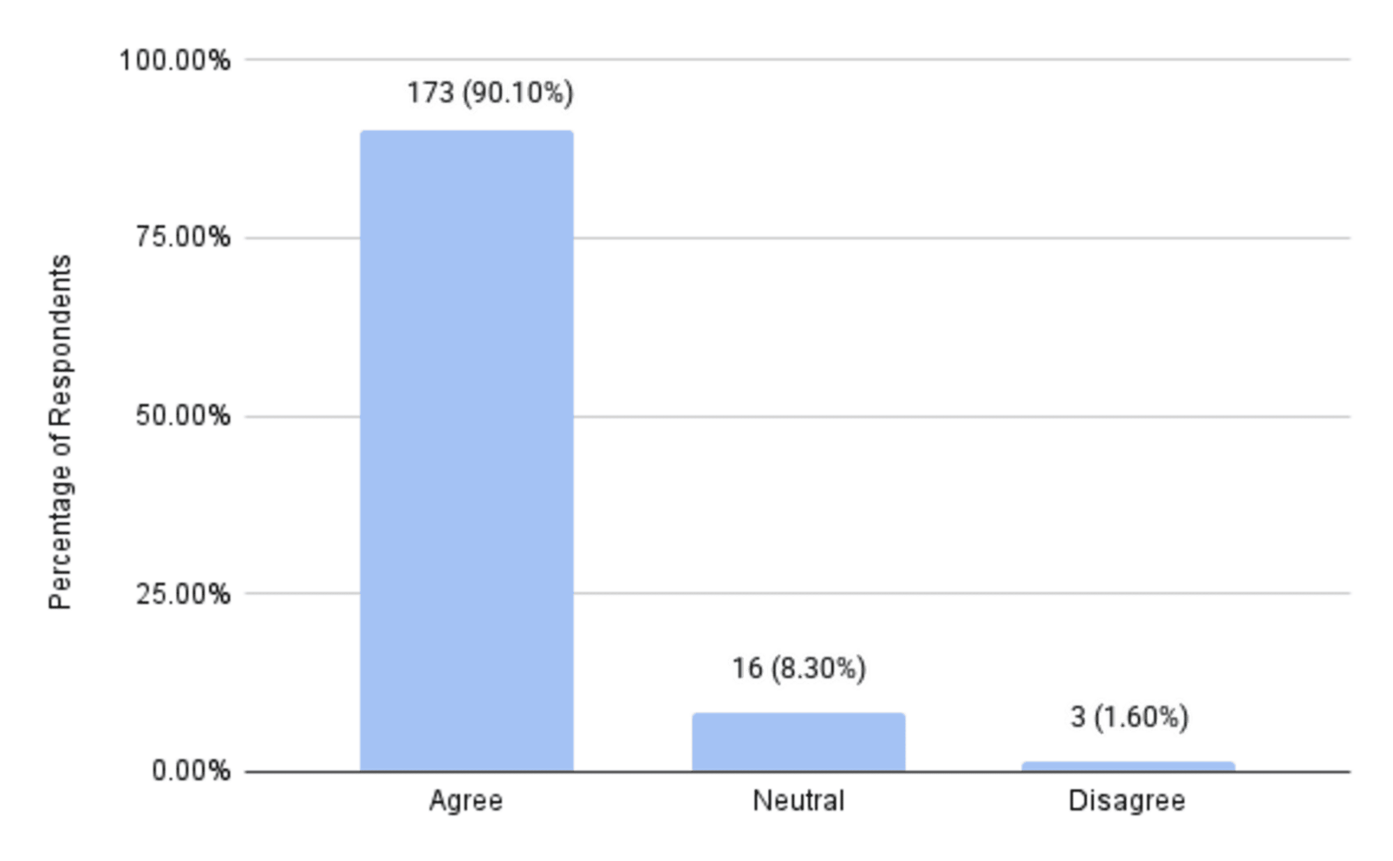
Participants’ opinions on the role of health educators in enhancing patient experience

A total of 173 (90.1%) participants agreed that health educators played an important role in enhancing patient experience, compared to the 3 (1.6%) participants who disagreed (Figure 3).

## Discussion

To the best of our knowledge, this is one of the few studies in Saudi Arabia to assess physicians’ and nurses’ awareness of the roles of health educators. The lack of comparable local studies highlights the importance of these findings. This study aimed to assess the awareness of physicians and nurses regarding the roles of health educators in healthcare settings. Based on our results, the gender distribution was nearly balanced. Physicians accounted for the majority of respondents at 63.02%, compared to 36.98% nurses. In terms of specialty, nearly half (47.80%) specialized in internal medicine, while the remaining participants were spread across various other specialties. 

In one study measuring the perception of Riyadh residents, more than 90% expressed a preference for having health educators in schools, believing that their presence would have a positive impact on students [11]. This is promising, as it indicates that the general population is aware of the roles and responsibilities of health educators and recognizes their positive impact on people's health.

A significant majority (69.30%) of participants demonstrated high levels of total knowledge and understanding regarding health educators' tasks, roles, and responsibilities. This is encouraging, as it suggests that most healthcare professionals recognize the importance of health educators within the hospital setting. However, the fact that 11.5% of participants scored low on their total knowledge and understanding of health educators' tasks, roles, and responsibilities indicates a need to investigate the reasons. Similarly, Almoayad et al. [5] found that half of healthcare professionals scored low in their understanding of the roles and responsibilities of health educators.

The significant link between experience and knowledge in our study suggests that early career professionals are well informed about the roles of health educators, with 70.30% of those with less than 5 years of experience and 84.78% of those with 5-10 years of experience demonstrating high knowledge of tasks, roles, and responsibilities. This may be attributed to new career professionals having been educated under more recent curricula that emphasize the role of health educators within the multidisciplinary healthcare team. However, when it comes to knowledge of the presence of health educators, the highest awareness was seen among those with over 15 years of experience (62.96%), while those with less than 5 years showed the lowest (35.64%). Thus, experienced healthcare professionals may be more aware of health educators' presence, likely due to greater familiarity and collaboration over time.

In our study, 90.30% of participants acknowledged the role of health educators in enhancing patient care, which aligns with a study conducted in healthcare centers in Riyadh, where 68% of participants reported satisfaction with health education services [12]. This similarity reflects the growing recognition of health educators’ role in patient care. Both this study and the Riyadh study show that health professionals’ acknowledgment aligns with satisfaction with health education roles.

Health promotion empowers individuals to manage their health [13] and is a key role of health educators. In a Saudi study, nearly 42% of 206 physicians and nurses were unaware of health promotion programs conducted in hospitals. Yet nearly 90% of physicians and nurses expressed willingness to participate in health promotion activities. Most interestingly, nearly 79% of physicians and nurses support mandating health promotion lectures and workshops for all healthcare providers by the hospital [14], reflecting their willingness to engage in interdisciplinary collaboration. The fact that 33.33% of our participants had never collaborated with a health educator, and 15.10% were unsure if they had, points to potential gaps in interprofessional collaboration. Moreover, around one-quarter of our participants (22.92%) reported low awareness regarding the presence of health educators in healthcare settings. Increasing the visibility and integration of health educators within healthcare teams may enhance interdisciplinary collaboration and improve patient outcomes. Thus, collaborative teamwork is crucial for promoting mutual understanding of the various responsibilities and roles held by different healthcare professionals, as it contributes to better patient outcomes, enhances team efficiency, and positively influences patient safety and satisfaction [15,16].

Only 51.24% of physicians and 52.11% of nurses reported having worked with health educators in a healthcare setting. This highlights a core issue, limited team collaboration, which may hinder the integration and effectiveness of health education in the healthcare setting. This points to a need for structured team collaboration, such as regular interdisciplinary meetings and clear role assignments, coordinated by hospitals and healthcare organizations.

Implications for hospital practice and recommendations

To address the identified gaps, hospitals should implement strategies to better integrate health educators into clinical teams. Increasing their visibility in patient care processes, such as involving them in clinical rounds and promoting their services to healthcare staff, could enhance interdisciplinary collaboration. 

Increasing awareness among healthcare providers about the roles of health educators could lead to better utilization of their skills, ultimately improving patient outcomes. Hospitals should align their health education strategies with the model of care by emphasizing the role of health educators in patient empowerment. This enhances prevention and engagement and helps physicians and nurses better understand the role of health educators [17].

Hospitals might also consider implementing regular interdisciplinary training sessions to increase staff awareness of health educators' contributions and promote collaborative practices. Targeted workshops and awareness campaigns could be organized to educate healthcare providers about the roles of health educators. 

Health educators should also utilize the Ministry of Health’s Health Awareness platform to provide trusted content and raise awareness among healthcare teams about their roles in promoting healthy behaviors [18]. Leveraging innovative educational tools, including mobile applications and virtual simulations, can further support both healthcare providers and patients in understanding treatment plans and post-discharge care instructions.

Strengths and limitations

This study has several strengths. First, it is one of the few studies conducted in Saudi Arabia to specifically assess physicians' and nurses' awareness of health educators' roles, filling a critical research gap in this area. The study benefits from a well-designed and validated questionnaire, which ensures the reliability and accuracy of the data collected. Moreover, understanding healthcare professionals' knowledge of health education roles can help identify and address barriers, ultimately improving collaboration within healthcare teams and enhancing patient outcomes. Lastly, support from SCFHS was essential in selecting top-tier physicians and nurses from diverse settings for data collection.

Despite its strengths, the study has notable limitations that must be acknowledged. The cross-sectional design limits the ability to establish causal relationships between variables. Also, due to the use of convenience sampling, the findings may not be generalizable to all healthcare providers. Moreover, most participants are from the middle region of Saudi Arabia, which restricts the generalizability and diversity of the results to other regions or healthcare settings. Additionally, a small sample size of 192 participants may not represent the whole community physicians and nurses in Saudi Arabia, yet it gives a brief perception of their awareness. The reliance on self-reported data through an online survey could also introduce some sort of bias, as participants may have overestimated their knowledge/awareness or misunderstood the questions. Finally, while the study identifies a few associations, it does not explore the underlying reasons for low awareness or reasons for lack of collaboration with health educators. Future research should address these limitations to enhance the external validity and depth of analysis.

## Conclusions

This study provides valuable insights into the current level of awareness and understanding of health educators' roles among physicians and nurses. While the high level of understanding among most healthcare professionals is encouraging, the findings reveal important areas for improvement in the integration and visibility of health educators within healthcare teams. The gap between understanding the roles of health educators and awareness of their presence in hospitals highlights a crucial issue that needs to be addressed.

The study reveals that experience plays a complex role in healthcare professionals' interactions with health educators. Early career professionals showed higher role knowledge, while those with more experience were more aware of health educators' presence.

## Appendices

**Table 8:** Study questionnaire (original Arabic version) Note: The ○ symbol indicates that participants could choose only one response.

Section number	Section name	Question	Response options
-	الموافقة والإقرار	أُقِر بأني فهمت الهدف من هذه الدراسة وأن المشاركة تطوعية، وباستطاعتي الانسحاب في أي وقت بدون أي عواقب وأعطي موافقتي على المشاركة في هذه الدراسة بالإجابة على الأسئلة.	〇 موافق 〇 غير موافق
1	القسم الإجتماعي	1.1 الرجاء تحديد النوع	〇 ذكر 〇 أنثى
		1.2 الرجاء تحديد العمر	〇 ١٨-٢٠ سنة 〇 ٢١-٢٥ سنة 〇 ٢٦-٣٠ سنة 〇 ٣١-٣٥ سنة 〇 ٣٦-٤٠ سنة 〇 ٤١-٤٥ سنة 〇 ٤٦-٥٠ سنة 〇 ٥١-٥٥ سنة 〇 ٥٦ سنة فما فوق
		1.3 الرجاء تحديد المهنة	〇 طبيب 〇 ممرض
		1.4 الرجاء تحديد التخصص مثال (تمريض/طبيب عيون او قلب او باطنة)	(سؤال مفتوح)
		1.5 الرجاء تحديد سنوات الخبرة	〇 أقل من ٥ سنوات 〇 ٥ إلى ١٠ سنوات 〇 ١١ إلى ١٥ سنة 〇 أكثر من ١٥ سنة
		1.6 الرجاء تحديد المستوى العلمي	〇 دبلوم 〇 بكالوريوس 〇 ماجستير 〇 الإقامة 〇 الزمالة
		1.7 الرجاء تحديد مكان عملك المعتاد	〇 العيادات الخارجية 〇 التنويم 〇 الطوارىء 〇 إدارة المستشفى 〇 آخرى: _________
		1.8 الرجاء تحديد منطقة العمل	〇 المنطقة الوسطى 〇 المنطقة الشمالية 〇 المنطقة الجنوبية 〇 المنطقة الشرقية 〇 المنطقة الغربية
2	قياس المعرفة	2.1 هل لديك معرفة عن التثقيف الصحي؟	〇 نعم 〇 لا
		2.2 هل يوجد في مكان عملك متخصصين تثقيف صحي؟	〇 نعم 〇 لا 〇 لا أعلم
		2.3 هل عملت في مكان العمل مع متخصصين تثقيف صحي؟	〇 نعم 〇 لا 〇 لا أذكر
		2.4 في حال كانت الإجابة بنعم، الرجاء تقدير المهام التي قمت بعملها مع متخصصي التثقيف الصحي	〇 نادرا 〇 بعض المهام 〇 معظم المهام 〇 جميع المهام 〇 لم أعمل مع التثقيف الصحي
		2.5 هل تعتقد ان تواجد متخصصين التثقيف الصحي امر مهم في المنشئات الصحية؟	〇 نعم 〇 لا 〇 لا أعلم
3	تواجد متخصصين التثقيف الصحي	3.1 برأيك يقتصر دور متخصصين التثقيف الصحي على التوعية فقط	〇 اتفق 〇 محايد 〇 لا اتفق
		3.2 برأيك يقتصر تواجد متخصصين التثقيف الصحي في العيادات الخارجية	〇 اتفق 〇 محايد 〇 لا اتفق
		3.3 برأيك يقتصر تواجد متخصصين التثقيف الصحي في اقسام التنويم فقط	〇 اتفق 〇 محايد 〇 لا اتفق
		3.4 برأيك يمكن القيام بمهام التثقيف الصحي من قبل اي متخصص صحي	〇 اتفق 〇 محايد 〇 لا اتفق
		3.5 برأيك تواجد متخصصين التثقيف الصحي يعتبر امر تكميلي وليس متطلب هام في المنشئات الصحية	〇 اتفق 〇 محايد 〇 لا اتفق
		3.6 مهام وأدوار متخصصين التثقيف الصحي ليست واضحة لي بشكل كامل	〇 اتفق 〇 محايد 〇 لا اتفق
		3.7 لا افضل العمل مع متخصصين التثقيف الصحي	〇 اتفق 〇 محايد 〇 لا اتفق
		3.8 يلعب متخصصين التثقيف الصحي دور هام في تحسين تجربة المرضى	〇 اتفق 〇 محايد 〇 لا اتفق
4	مهام وأدوار ومسؤوليات متخصصين التثقيف الصحي	4.1.1 متخصصين التثقيف الصحي لديهم مهارات البحث	〇 اتفق 〇 محايد 〇 لا اتفق
		4.1.2 لدى متخصصين التثقيف الصحي مهارات حل المشكلات	〇 اتفق 〇 محايد 〇 لا اتفق
		4.1.3 لدى متخصصين التثقيف الصحي فهم جيد للأمراض والاضطرابات الشائعة	〇 اتفق 〇 محايد 〇 لا اتفق
		4.1.4 لدى متخصصين التثقيف الصحي القدرة على التواصل بشكل فعال مع المرضى والأسر	〇 اتفق 〇 محايد 〇 لا اتفق
		4.1.5 لدى متخصصين التثقيف الصحي القدرة على تقديم الدعم النفسي للمرضى والأسر	〇 اتفق 〇 محايد 〇 لا اتفق
		4.1.6 لدى متخصصين التثقيف الصحي القدرة على تطوير وتنفيذ برامج التثقيف الصحي	〇 اتفق 〇 محايد 〇 لا اتفق
		4.2.1 يلعب متخصصي التثقيف الصحي دور مهم في تعزيز صحة الفرد والمجتمع.	〇 اتفق 〇 محايد 〇 لا اتفق
		4.2.2 يلعب متخصصين التثقيف الصحي دورًا مهمًا في مساعدة المرضى على إدارة صحتهم بشكل مستقل	〇 اتفق 〇 محايد 〇 لا اتفق
		4.2.3 يلعب متخصصين التثقيف الصحي دورًا مهمًا في تقليل العبء على النظام الصحي	〇 اتفق 〇 محايد 〇 لا اتفق
		4.3.1 متخصصين التثقيف الصحي لديهم القدرة على العمل بشكل مستقل	〇 اتفق 〇 محايد 〇 لا اتفق
		4.3.2 متخصصين التثقيف الصحي لديهم القدرة على العمل تحت الضغط.	〇 اتفق 〇 محايد 〇 لا اتفق
		4.3.3 متخصصين التثقيف الصحي لديهم القدرة على التكيف مع التغييرات في الرعاية الصحية (تحول القطاع الصحي، نموذج الرعاية الصحي الحديث).	〇 اتفق 〇 محايد 〇 لا اتفق

**Table 9:** Study questionnaire (translated English version) Note: The ○ symbol indicates that participants could choose only one response.

Section number	Section name	Question	Response options
-	Consent and acknowledgment	I acknowledge that I understand the purpose of this study and that participation is voluntary, and I can withdraw at any time without any consequences. I give my consent to participate in this study by answering the questions.	〇 Agree 〇 Disagree
1	Sociodemographic characteristics	1.1 Gender	〇 Male 〇 Female
		1.2 Age	〇 18-20 years old 〇 21-25 years old 〇 26-30 years old 〇 31-35 years old 〇 36-40 years old 〇 41-45 years old 〇 46-50 years old 〇 51-55 years old 〇 56 years and older
		1.3 Profession	〇 Physician 〇 Nurse
		1.4 Specialty (e.g., nurse/doctor ophthalmologist, cardiologist, internal medicine, etc.)	(Open-ended)
		1.5 Years of experience	〇 Less than 5 years 〇 5 to 10 years 〇 11 to 15 years 〇 Over 15 years
		1.6 Educational level	〇 Diploma 〇 Bachelor’s degree 〇 Master’s degree 〇 Residency 〇 Fellowship
		1.7 Usual place of work	〇 Outpatient clinic 〇 Inpatient department 〇 Emergency room 〇 Hospital administration 〇 Other: _________
		1.8 Region	〇 Middle region 〇 Northern region 〇 Southern region 〇 Eastern region 〇 Western region
2	General knowledge	2.1 Do you know about health education?	〇 Yes 〇 No
		2.2 Do you have health educators at your place of work?	〇 Yes 〇 No 〇 I don’t know
		2.3 Have you ever worked with health educators?	〇 Yes 〇 No 〇 I don’t remember
		2.4 If yes, please estimate the number of tasks conducted with health educators.	〇 Rarely 〇 Some tasks 〇 Most tasks 〇 All tasks 〇 I have not worked with health educators
		2.5 Do you believe that the presence of health educators is important in health facilities?	〇 Yes 〇 No 〇 I don’t know
3	Presence of health educators	3.1 In your opinion, the role of a health educator is limited to increasing awareness only.	〇 Agree 〇 Neutral 〇 Disagree
		3.2 In your opinion, the place of work for a health educator is limited to outpatient clinics.	〇 Agree 〇 Neutral 〇 Disagree
		3.3 In your opinion, the place of work for a health educator is limited to inpatient departments.	〇 Agree 〇 Neutral 〇 Disagree
		3.4 In your opinion, the tasks of a health educator can be conducted by any health professional.	〇 Agree 〇 Neutral 〇 Disagree
		3.5 In your opinion, the presence of health educators is a complementary matter, not an important requirement in health facilities.	〇 Agree 〇 Neutral 〇 Disagree
		3.6 The tasks and roles of health educators are not entirely clear to me.	〇 Agree 〇 Neutral 〇 Disagree
		3.7 I would not prefer working with a health educator.	〇 Agree 〇 Neutral 〇 Disagree
		3.8 Health educators play an important role in enhancing patient experience.	〇 Agree 〇 Neutral 〇 Disagree
4	Tasks, roles, and responsibilities of health educators	4.1.1 Health educators have research skills.	〇 Agree 〇 Neutral 〇 Disagree
		4.1.2 Health educators have problem-solving skills.	〇 Agree 〇 Neutral 〇 Disagree
		4.1.3 Health educators have a good understanding of common diseases and disorders.	〇 Agree 〇 Neutral 〇 Disagree
		4.1.4 Health educators have the ability to effectively communicate with patients and their families.	〇 Agree 〇 Neutral 〇 Disagree
		4.1.5 Health educators have the ability to provide emotional support to patients and their families.	〇 Agree 〇 Neutral 〇 Disagree
		4.1.6 Health educators have the ability to develop and implement health education programs.	〇 Agree 〇 Neutral 〇 Disagree
		4.2.1 Health educators play an important role in promoting the health of individuals and communities.	〇 Agree 〇 Neutral 〇 Disagree
		4.2.2 Health educators play an important role in helping patients take control of and manage their health independently.	〇 Agree 〇 Neutral 〇 Disagree
		4.2.3 Health educators play an important role in reducing the burden on the health system.	〇 Agree 〇 Neutral 〇 Disagree
		4.3.1 Health educators have the ability to work independently.	〇 Agree 〇 Neutral 〇 Disagree
		4.3.2 Health educators have the ability to work under pressure.	〇 Agree 〇 Neutral 〇 Disagree
		4.3.3 Health educators have the ability to adapt to changes in health care (health sector transformation, modern healthcare model, etc.).	〇 Agree 〇 Neutral 〇 Disagree
